# Optimizing Interventions for Equitability: Some Initial Ideas

**DOI:** 10.1007/s11121-024-01644-3

**Published:** 2024-01-31

**Authors:** Jillian C. Strayhorn, David J. Vanness, Linda M. Collins

**Affiliations:** 1 Department of Social and Behavioral Sciences, School of Global Public Health, New York University, New York, USA; 2 Department of Health Policy and Administration, Pennsylvania State University, Pennsylvania, USA

**Keywords:** Intervention optimization, Multiphase optimization strategy, Health equity, Value efficiency

## Abstract

Interventions (including behavioral, biobehavioral, biomedical, and social-structural interventions) hold tremendous potential not only to improve public health overall but also to reduce health disparities and promote health equity. In this study, we introduce one way in which interventions can be optimized for health equity in a principled fashion using the multiphase optimization strategy (MOST). Specifically, we define intervention equitability as the extent to which the health benefits provided by an intervention are distributed evenly versus concentrated among those who are already advantaged, and we suggest that, if intervention equitability is acknowledged to be a priority, then equitability should be a key criterion that is balanced with other criteria (effectiveness overall, as well as affordability, scalability, and/or efficiency) in intervention optimization. Using a hypothetical case study and simulated data, we show how MOST can be applied to achieve a strategic balance that incorporates equitability. We also show how the composition of an optimized intervention can differ when equitability is considered versus when it is not. We conclude with a vision for next steps to build on this initial foray into optimizing interventions for equitability.

## Overview

Behavioral, biobehavioral, biomedical, and social-structural interventions (hereafter referred to simply as interventions) hold tremendous promise for prevention of disease and promotion of health and well-being. A few examples (out of many possibilities) include prevention of substance use (e.g., Hill et al., 2023), internalizing symptoms (e.g., Brincks et al., 2023), and HIV (Gwadz et al., 2022). Given the important and ubiquitous role that interventions play in public health, interventions should be expected to play a correspondingly important role in achieving health equity and eliminating health disparities. At the very least, interventions should not exacerbate existing health disparities; ideally, they should be designed specifically to reduce or eliminate health disparities.

In this article, we offer initial ideas for using intervention optimization via the multiphase optimization strategy (MOST; e.g., Collins, 2018; Collins et al., 2016, 2021) as a principled framework for designing interventions so as to reduce health disparities and promote health equity. In optimization, competing considerations (or “criteria”) are balanced strategically in deciding which intervention components and component levels to include in an intervention. To date, effectiveness, affordability (defined in terms of any finite resource, including money, participant time, and so on), scalability, and efficiency have been suggested as criteria to be used in MOST, usually with effectiveness balanced against the other criteria. For example, a reduction in expected intervention effectiveness might be traded to achieve affordability, thereby improving intervention reach and, ultimately, public health impact.

However, this strategic balancing has generally overlooked health equity. As we describe further below, alternative combinations of intervention components (or component levels) may plausibly have different effects for different groups of people, depending, for example, on some level of relative advantage versus disadvantage. This means that the decision-makers who select an optimized intervention may face the challenge of weighing equity against other criteria. The purpose of this article is to explore how equity can be explicitly included as a criterion to be balanced along with effectiveness, affordability, scalability, and efficiency in the optimization of an intervention.

We begin by briefly introducing intervention optimization and MOST, and we discuss health equity in relation to MOST. We then introduce a hypothetical equity case study in which we demonstrate the use of MOST to optimize by balancing equity with other important criteria. We conclude by discussing additional considerations and potential future directions. We emphasize that we see this article as an initial foray and make no claims that it is a complete treatment of this complex topic; in fact, much remains to be done. We hope this article helps to generate a broader conversation about the use of intervention optimization as a methodological framework for designing equitable interventions.

## Brief Introduction to Intervention Optimization and MOST

### A Critique of the Classical Treatment Package Approach

For much of the history of intervention science, interventions have been developed and evaluated using what we will call the classical treatment package approach (CTPA; also referred to as the “treatment package strategy” by Kazdin (1979)). In this approach, a set of intervention components is identified and assembled a priori into a treatment package. The treatment package as a whole is then evaluated by conducting an evaluation randomized controlled trial (ERCT) to compare it to a suitable control. Collins and colleagues (Collins et al., 2021) have critiqued the CTPA on numerous grounds. Three general themes emerge in these critiques. First, because the performance of individual intervention components, and whether they interact, is not directly assessed in the CTPA, it is usually unclear whether there are weak parts of interventions that would benefit from amendment or replacement (and if so, which parts). This inhibits the kind of programmatic, iterative improvement over time that has occurred in nearly every other field (e.g., automobiles, computers and software) over the past hundred years or so. Second, in the CTPA, *efficiency* (which we define here as “extent to which the intervention produces preferred outcomes without resource waste, relative to alternative interventions”) is not considered empirically. An intervention cannot be considered efficient if some alternative version of it—say, with an inert or counterproductive component removed—can be expected to produce better outcomes at the same or lower cost. Inefficient intervention packages squander resources that could and should be redeployed elsewhere, i.e., to other efforts that will improve public health and health equity. Third, in the CTPA, *affordability* (“extent to which the intervention is deliverable within budget, and offers a good value;” Collins et al., 2021, p. 2000) and *scalability* (“extent to which the intervention is implementable in the intended setting with no need for ad hoc modifications;” Collins et al., 2021, p. 2000) are considered only after the intervention package has been constituted and evaluated. At this point, the only route to achieving affordability and/or scalability is usually removal of components, without any empirical basis for selection of components so as to have the least deleterious effect on key outcomes.

Here, we extend this critique to note that in the CTPA, *equitability*, like affordability and scalability, is usually not considered until after the intervention package has been constituted and evaluated. Based on the Robert Wood Johnson Foundation’s definition of health equity (Braveman et al., 2017), we define equitability of an intervention as the *extent to which the health benefits provided by an intervention are distributed evenly*, *such that all participants have a fair and just opportunity to achieve the desired outcome of the intervention*. In our view, progress toward achieving health equity cannot be made by developing and evaluating interventions using the CTPA. Instead, we suggest that investigators optimize interventions prior to evaluation, including equitability as one of the considerations that is balanced in the process.

### The Concept of Intervention Optimization

It is foundational to our thinking that although intervention effectiveness is a critically important consideration in achieving public health impact, ultimately net public health impact is even more important. An intervention that is effective but not affordable or scalable will never be widely implemented, and therefore, its net public health impact will be nearly zero. Opportunity costs diminish the net public health impact of inefficient interventions. Intervention optimization has been defined as a process of identifying interventions that strategically balance effectiveness with affordability, scalability, and/or efficiency (Collins et al., 2021). An intervention optimization perspective on health equity extends this thinking to emphasize that an intervention that is effective overall but not equitable—for example, because benefits are concentrated among those who are already advantaged—will also have diminished public health impact, and possibly, even worsen health disparities. We propose that intervention optimization can be defined as the process of identifying the intervention that strategically balances intervention *effectiveness* overall with intervention *equitability*, *affordability*, *scalability*, and/or *efficiency*.

### Brief Introduction to Intervention Optimization Using MOST

MOST consists of three phases: preparation, optimization, and evaluation. In the preparation phase, the investigator begins by deriving a detailed conceptual model of the health outcome(s) to be intervened on and identifies a set of intervention components that are candidates for inclusion in the intervention. The decision about whether or not to include these components will be made based on empirical results obtained in the next phase. Decision-making will depend on the optimization objective, which defines the desired strategic balance of effectiveness and additional criteria (e.g., Is affordability relevant? If so, what is the available budget?). In the next phase of MOST, the investigator optimizes the intervention. A necessary part of this process is conducting one or more optimization randomized controlled trials (ORCTs). There are many experimental designs to choose from for the ORCT, all of which provide information on the individual and combined performance of intervention components. The results of the ORCT(s) are used to inform careful decision-making about the candidate intervention components, with the ultimate goal of identifying the combination of components that best meets the optimization objective. In other words, the results of the ORCT(s) are used to confront tradeoffs among the competing considerations laid out in the optimization objective. Below, we illustrate such tradeoffs in more detail, and with explicit consideration of intervention equitability as one competing criterion, using a hypothetical case study. In the final phase of MOST, the optimized intervention is evaluated as a package in a classical ERCT. A comprehensive introduction to MOST can be found in Collins (2018).

## A Hypothetical Case Study

### Introduction to the Case Study

#### The Motivating Scenario

Suppose an investigator is applying MOST to develop and optimize an intervention to improve medication adherence among people living with HIV. This investigator has identified some candidate intervention components that they hypothesize will contribute to more successful medication adherence. However, because of glaring health disparities in HIV treatment (Gwadz et al., 2017), the investigator wishes to identify an intervention that is effective overall but also with as much equitability as possible—and certainly with no disparity-widening effects. Furthermore, since the investigator also recognizes that the monetary resources for intervention delivery are scarce, they wish to identify an intervention that is efficient in its use of the available resources. This means that three criteria emerge as priorities in this scenario: overall intervention effectiveness, equitability, and efficiency.

#### Acknowledging Some Simplifying Assumptions

The case study we select as the logical starting point for this illustration of our proposed methods is simplified in certain key ways. First, we assume the decisions about which components to include in the optimized intervention are to be based on a single continuous outcome variable representing medication adherence. Second, we assume that the investigator is optimizing a fixed (versus adaptive) intervention and that they use a prototypical 2^*k*^ factorial design for their ORCT. Third, we assume that the hypothetical intervention is single-level versus multilevel, with all candidate components delivered at the individual level. In the “Discussion,” we return to these assumptions as we lay out a vision for the extension of the methods proposed here.

### The Case Study ORCT

Suppose our case study investigator has identified four candidate intervention components (based loosely on components from Gwadz et al., 2017): Motivational Interviewing, Peer Support, a Navigator, and Skill-Building Sessions. Though all components are hypothesized to contribute to adherence generally, the investigator also hypothesizes Motivational Interviewing may not be as effective for individuals with more social disadvantage. With this in mind, the team designs the Navigator component to help individuals from all levels of relative advantage versus disadvantage access and engage in care, hypothesizing further that the presence versus absence of the Navigator could help overcome important systemic barriers to healthcare access that would otherwise make certain alternative interventions (e.g., those that contain Motivational Interviewing) less effective for individuals with less advantage. The investigator’s optimization objective is to choose an optimized intervention that strategically balances intervention effectiveness (in terms of better medication adherence) with equitability (in terms of the distribution of intervention benefits across participants) and efficiency (in terms of the use of available monetary resources for intervention delivery).

With this optimization objective in mind, the investigator chooses a 2^4^ factorial design for their ORCT and operationalizes the candidate components as factors with the levels “On” versus “Off,” such that components are absent or present in the 2^4^ = 16 alternative interventions (composed of the 16 alternative combinations of factor levels). One of the great advantages of the factorial ORCT (e.g., Collins et al., 2009, 2014) is the wealth of empirical information that can be obtained. In this example, this includes four main effects and up to 11 interaction effects. A main effect for any given factor indicates that the factor demonstrates an effect on average across all combinations of levels of the remaining three factors. A two-way interaction effect indicates that the effect of one factor differs depending on the level to which a second factor is set. The outcome variable is a hypothetical continuous measure of medication adherence, and the costs associated with delivering the alternative interventions are measured in US dollars. Everything we present assumes the ORCT involves a sample sufficiently representative to enable valid conclusions to be drawn about equity across various levels of relative advantage versus disadvantage; we return to this in the “Discussion.”

The latest recommended practice for decision-making about the composition of optimized interventions in MOST involves using a posterior expected value approach (Strayhorn et al., 2023a). In this approach, which incorporates methods from Bayesian decision science (e.g., Claxton, 1999), empirical information from all of the main and interaction effects is used to estimate an expected outcome for each alternative intervention (i.e., each combination of factor levels). We assume in this case study that a large expected outcome is better; if the investigators were choosing an optimized intervention based on overall effectiveness only, they would choose the alternative intervention that is associated with the largest expected outcome in their ORCT. However, since the investigator’s objective is to strategically balance overall effectiveness with equitability and efficiency, the choice of optimized intervention may or may not be the intervention associated with the largest expected outcome in the ORCT. We have elsewhere proposed a decision-making framework, decision analysis for intervention value efficiency (DAIVE), to facilitate multi-criteria decision-making using a posterior expected value approach in MOST (Strayhorn et al., 2023b). Next, we introduce our simulated case study data and then demonstrate the use of DAIVE for decision-making about the composition of the optimized intervention—first without consideration of equitability and then with.

### Methods for Simulating Case Study ORCT Data

We generated a simulated ${2}^{4}$ factorial ORCT based on the hypothetical scenario just described with 16 alternative interventions $t$ comprising up to four candidate intervention components. We coded the four candidate components as two-level factors $\left\{A,B,C,D\right\}$ with the levels “On” vs “Off” and defined a single outcome variable (medication adherence). In our notation, we identify alternative interventions by the factors set to “On” in a given intervention; for example, intervention AB indicates that factors *A* and *B* are set to “On,” meaning that the components associated with *A* and *B* are included in that intervention, but components associated with *C* and *D* are not. We then defined groups $g\in \{\mathrm{1,2},\mathrm{3,4},5\}$ based on quintiles of a randomly-generated systemic advantage measure for each individual $i$, ${S}_{i}\sim U\left(\mathrm{0,1}\right),$ and assigned $N=10$ individuals in each group to each of the ${2}^{4}=16$ alternative interventions, for a total *N* = 800. (We acknowledge that our illustration is done with a sample size that may seem unrealistically large; we return to this in the “Discussion.”) For our purposes, we let $S$ quantify continuous disadvantage-to-advantage generally, meaning that more or less relative advantage could be for any number of reasons.

Starting with the “minimal intervention” $$Min$$, in which all factors are set to “Off,” we defined average treatment effects (assumed to be changes in the outcome relative to baseline) to produce decision-making tradeoffs, consistent with the hypothetical scenario. For simplicity, we assigned $$Min$$ an effect (relative to baseline) of zero. We then let factor *A* represent Motivational Interviewing, and we gave *A* an expected effect that was linearly increasing in advantage measure $${S}_{i}$$, $${\mu }_{A}=0.5+0.5{S}_{i}$$, such that individuals at the lowest level of relative advantage ($${S}_{i}=0)$$ had an expected effect on the outcome of 0.5, while individuals at the highest level of relative advantage ($${S}_{i}=1)$$ had an expected effect of 1.0. Next, we let factor *B* represent Peer Support, and we gave *B* an expected effect that was also linearly increasing in advantage, $${\mu }_{B}={S}_{i}$$. We let factor *C* represent the Navigator, and we let *C* have no effect on its own ($${\mu }_{C}=0$$) but be involved in an interaction with factor *B*, offsetting *B*’s lower performance for individuals with lower advantage, such that $${\upmu }_{{\text{BC}}}={\mu }_{B}+{\mu }_{C}+(1-{S}_{i})$$ = 1. Finally, we let factor *D* represent Skill-Building Sessions, and we gave *D* an expected effect that was decreasing in relative advantage $${\mu }_{D}=1-{S}_{i}$$, such that *D* had an expected effect of 0 for the most advantaged individual and 1 for the least advantaged individual. In other words, Motivational Interviewing (*A*) and Peer Support (*B*) work less well for those with relatively less advantage; Navigator (*C*) has no effect on average but offsets the poorer performance of Peer Support (*B*) for those with relatively less advantage; and Skill-building Sessions (*D*) work better for individuals with relatively less advantage. For simplicity, we generated data such that no interactions were expected other than the one involving factors *B* (Peer Support) and *C* (Navigator); therefore the expected effects for interventions that did not include both Peer Support and Navigator are simply the sums of the expected effects for any involved factors set to “On.”

We labeled the 16 alternative interventions using letters, based on which factors were set to “On” in a given intervention. For example, intervention $t={\text{AB}}$ had factors *A* and *B* (representing the hypothetical Motivational Interviewing and Peer Support components) set to “On” and factors *C* and *D* (representing the hypothetical Navigator and Skill-Building Sessions components) set to “Off.” Outcomes ${Y}_{{\text{i}}}$ for all individuals $i$ were generated from a normal distribution centered on the intervention effect, ${Y}_{{\text{i}}}\sim N({\mu }_{{\text{t}}}({S}_{{\text{i}}}),1)$. Finally, we gave the “On” versus “Off” levels of our four factors delivery costs, which we selected arbitrarily as follows: ${C}_{A}=100, {C}_{B}=125, {C}_{C}=200, {C}_{D}=250$. We assumed that the cost for a given alternative intervention was the sum of the costs for any factors set to “On” in that particular intervention.

Consistent with a posterior expected value approach to optimization decision-making (Strayhorn et al., 2023a, b), we used CmdStan (version 2.30.0) via cmdstanr (version 0.5.2) for rstan (Stan Development Team, 2020) and brms (2.18.0) (Bürkner, 2021) packages in the R (version 4.2.1) statistical environment (R Core Team, 2021) to obtain estimates of posterior expected outcomes under each alternative intervention using Markov Chain Monte Carlo. We specified relatively parsimonious linear regression models that included main effects and two-way interactions, with default priors. We fit separate models for each relative advantage quintile group $$g$$ ($$N=160)$$ and also fit a model for the full sample ($$N=800)$$.

### Results, Part I: Balancing Effectiveness and Efficiency Using DAIVE

**Fig. 1:**
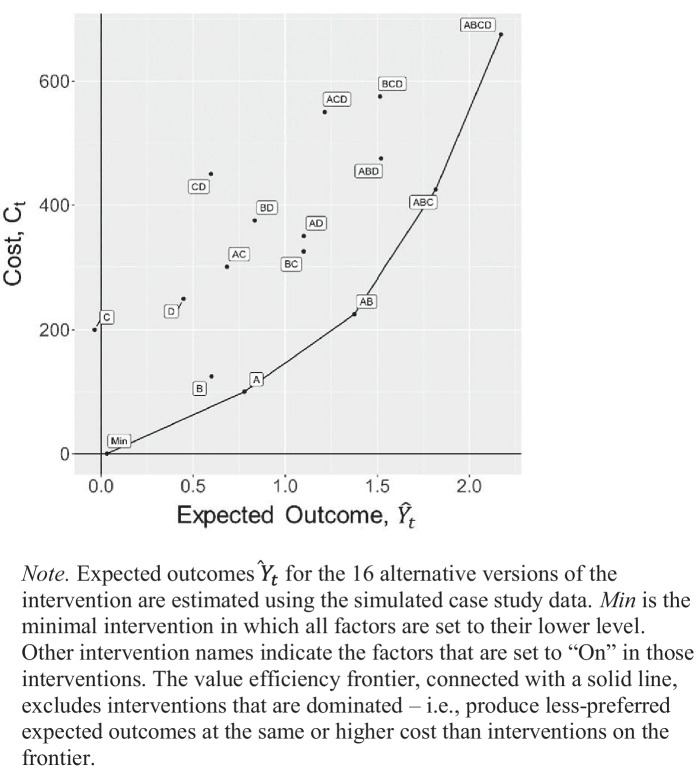
Cost-expected outcome scatterplot and value efficiency frontier for the simulated factorial ORCT

To introduce the use of DAIVE (Strayhorn et al., 2023b) in intervention optimization decision-making, we begin without considering equitability as a criterion, instead balancing only effectiveness and efficiency. DAIVE provides investigators who are interested in balancing effectiveness and efficiency with an x–y scatterplot of cost (*C*_t_) versus expected outcome (${\widehat{Y}}_{{\text{t}}}$), with expected outcomes estimated using data from a given ORCT. One such scatterplot based on the simulated case study data is shown in Fig. 1. In this plot, there are 16 points, one for each of the alternative interventions under consideration in the case study (i.e., from *Min* to intervention ABCD). Examining the relative positions of the points on the plot can give a general sense of which are more effective (i.e., larger in their expected outcome) and which are less costly. Efficiency, meanwhile, is achieved by the alternative interventions that are expected to produce more value (in this case, in terms of a larger expected outcome ${\widehat{Y}}_{t}$) for less cost; these “value efficient” alternatives fall on the lower-right convex hull (connected with a solid line in Fig. 1 and henceforth referred to as a “value efficiency frontier”). All other alternative interventions (not on this value efficiency frontier) are “dominated” in the sense that they cost more to achieve less-preferred expected outcomes. By default, the value efficiency frontier begins with the least costly alternative (in the case study, the minimal intervention *Min*).

The investigator who wants to strategically balance effectiveness and efficiency would select an optimized intervention from among the set of value efficient alternatives. In other words, the dominated alternatives are removed from consideration, whereas interventions on the value efficiency frontier are contenders for selection as the optimized intervention, depending on the anticipated willingness-to-pay to achieve more-preferred outcomes. In most (if not all) applications of MOST, the investigator is not the payer, so they anticipate the eventual payer(s) who, for example, will be responsible for funding the implementation of an eventual intervention. The willingness-to-pay represents the resources (e.g., US dollars for intervention delivery) an eventual payer is willing to spend (or reallocate from other useful programs) in order to accomplish a unit improvement in the outcome (e.g., medication adherence).

In the case study, there are four interventions on the value efficiency frontier (other than *Min*, which is the default starting point): intervention A, intervention AB, intervention ABC, and intervention ABCD. To choose an optimized intervention, the investigator considers pairs of value efficient alternatives systematically to determine whether the added value of the more expensive in the pair would be worth its added cost. For example, an investigator who does not perceive the difference in expected outcomes between ABCD and ABC to be worth the added cost for ABCD versus ABC might choose ABC as the optimized intervention. Usually, this reflects the acknowledgment of opportunity costs, or the idea that the resources a payer saves by choosing, say, ABC over ABCD could potentially be allocated elsewhere, e.g., to other useful programs. By contrast, an investigator who anticipates a larger willingness-to-pay might choose the intervention that maximizes the expected outcome, intervention ABCD, as the optimized intervention. The balancing of effectiveness and efficiency, again, is strategic; it is intended to reflect context, including specific priorities and resource-availability.

### Results, Part II: Balancing Effectiveness, Efficiency, and Equitability Using DAIVE

We now return to the original objective of strategically balancing effectiveness, efficiency, and equitability, illustrating one possible way of going about this using DAIVE. We present this in three steps: first, converting two distinct criteria, effectiveness and efficiency, into a single criterion, “net health value”; second, quantifying intervention equitability; and third, strategically balancing net health value and equitability.

#### Step 1: Converting Effectiveness and Efficiency into Net Health Value

In this step, we make use of a relationship well-known to cost-effectiveness researchers (Stinnett & Mullahy, 1998) converting the two dimensions of costs and outcome gains for an intervention $t\in \{1,\dots ,T\}$ to a unidimensional comparative measure of net health value:

1 $$NH{V}_{t}\left(\lambda \right)=\lambda \times {\widehat{Y}}_{t}-{C}_{t}$$

where $\lambda$ is the willingness-to-pay, ${\widehat{Y}}_{{\text{t}}}$ is the estimated expected outcome for intervention $t$, and ${C}_{{\text{t}}}$ is the cost for intervention $t$. Above, when the investigator was balancing only effectiveness and efficiency, we acknowledged the concept of willingness-to-pay somewhat vaguely, as a systematic determination of whether a certain outcome gain was “worth” a certain added cost. Now, for the calculation of predicted net health values, willingness-to-pay has to be quantified more explicitly, since net health value reflects efficiency determinations that depend on willingness-to-pay (such that, at higher willingness-to-pay, more costly interventions may be considered to be worth their associated costs). As such, $\lambda$ is a representation of the decision-maker’s perceived economic opportunity costs based on their relative preferences for spending limited resources to improve outcome $Y$ or using those resources for the next best alternative use. All else equal, the more important outcome $Y$ is relative to other outcomes, and the more resources the decision-maker has available, the higher the willingness-to-pay. The steps we take to define representative willingness-to-pay thresholds of different sizes (using the results of our simulated ORCT and the arbitrarily-chosen delivery costs) are detailed in the Technical Appendix; following these steps, we chose the following set of willingness-to-pay thresholds $\left\{\$170,\$330,\$580,\$830\right\}$, representing the key points at which decision-making about the optimized intervention might differ, reflecting different “strategies” for the strategic balancing of effectiveness and efficiency. We proceed by calculating predicted net health values for all of the alternative interventions (i.e., not only the value efficient set *M* but all 16 alternatives) four times, at each identified willingness-to-pay.

#### Step 2: Quantifying Intervention Equitability

For this initial case study, we propose to quantify, for each alternative intervention in the ORCT, the distribution of health gains among individuals with different levels of advantage, approached in terms of a concentration so as to standardize across different discrete groups. We will accomplish this by modifying existing concentration metrics such as the health concentration curve and concentration index (Wagstaff et al., 1991) As noted above, we let $S$ be a continuous measure of systemic advantage, assumed to be increasing in advantage, and we let individuals be sorted by $S$, from least advantaged to most advantaged, and then stratified into a finite number of groups $g\in \left\{1,\dots ,G\right\}$(increasing in $S$) in order to calculate the health concentration curve and concentration index. We let ${R}_{g}\in (\mathrm{0,1}]$ be the highest individual percentile rank of $S$ in each group, with ${R}_{0}\equiv 0$ by construction. For example, suppose that for group $g=1$, ${R}_{1}=0.2$. Then, group 1 comprises all individuals from the lowest-ranked through the 20th percentile of $S$. Next, let ${\widehat{Y}}_{{\text{t}},{\text{g}}}$ be the empirically estimated measure of expected health outcome gains (relative to pre-intervention baseline) for individuals in group $g$ assigned to intervention $t\in \{1,\dots ,T\}$. Although we at various points optimistically use the term “gains,” we note here that it is also possible that interventions may unintentionally worsen outcomes of interest; in such cases, the gains may take on negative values. We propose ruling out interventions with zero or negative cumulative expected health gains over the entire population, $\sum_{g=1}^{G}{w}_{{\text{g}}}{\widehat{Y}}_{{\text{t}},{\text{g}}}\le 0$ (where ${w}_{{\text{g}}}$ is a weight corresponding to the proportion of the population in group $g$. Under this restriction, it is possible for gains in one or more groups to be negative as long as gains in other groups are large enough such that the total population health gain is positive.

To quantify the overall performance of the alternative interventions in terms of equitability, we first define concentrations curves using expected outcomes estimated in the ORCT. The height of the concentration curve of cumulative expected health gains for intervention $t$ at systemic advantage rank ${R}_{{\text{g}}}$ is

2 $${h}_{t}\left({R}_{g}\right)= \frac{\sum_{j=1}^{g}{\widehat{Y}}_{t,j}}{\sum_{j=1}^{G}{\widehat{Y}}_{t,j}}$$

where ${h}_{{\text{t}}}(0)\equiv 0$ by definition and ${h}_{{\text{t}}}\left(1\right)=1$ by construction for all $t$. If health outcome gains are independent of systemic advantage, then ${h}_{{\text{t}}}\left({R}_{{\text{g}}}\right)={R}_{{\text{g}}}$, and the concentration curve lies on the 45° line. If an alternative intervention offers more benefit to those with lower relative advantage, then the concentration curve will primarily be above the 45° line; if an intervention offers more benefit to those with more relative advantage, the concentration curve will primarily be below the 45° line.

We then use these concentration curves to define a health gain concentration index for each alternative intervention. For intervention $t$, the health gain concentration index ${H}_{{\text{t}}}$ is two times the area between the concentration curve and the 45° line:

3 $${H}_{t}=\sum_{g=1}^{G}\left({R}_{g}-{R}_{g-1}\right) \left(\left({R}_{g-1}-{h}_{t}\left({R}_{g-1}\right)\right)+\left({R}_{g}-{h}_{t}\left({R}_{g}\right)\right)\right)$$

The health gain concentration index ${H}_{{\text{t}}}$ is positive when health gains for intervention $t$ are concentrated among individuals with high advantage, negative when gains are concentrated among those with low advantage, and zero when health gains are uncorrelated with advantage. For readers familiar with the standard health concentration curve and index (Wagstaff et al., 1991), we note that in our modification the health gain concentration index is not necessarily bounded between $[-\mathrm{1,1}$]; this is due to the possibility of more negative health gains from iatrogenic interventions. Because we prefer to have our measure of concentration reversed, such that higher values indicate interventions that are equity-enhancing, we finally let our measure of intervention value equitability ${Q}_{{\text{t}}}$ be defined as:

4 $${Q}_{t}=-{H}_{t}$$

**Fig. 2:**
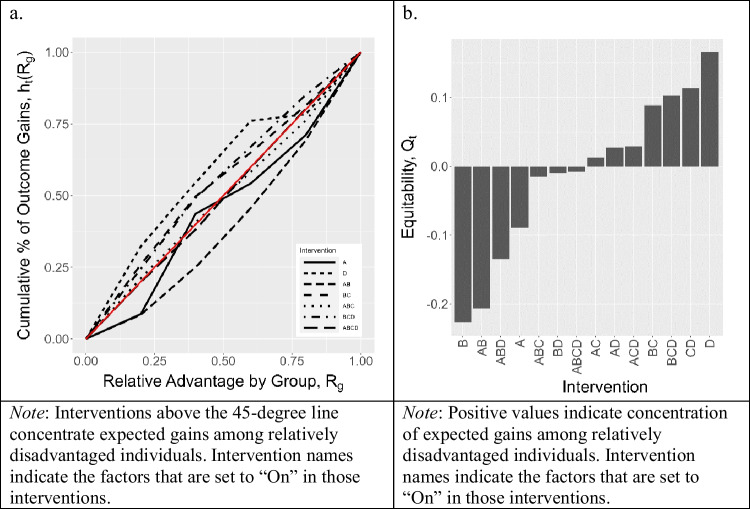
Concentration curves for selected interventions (**a**, left) and equitability values for all interventions (**b**, right)

Figure 2a and b depicts select concentration curves $${h}_{{\text{t}}}\left({R}_{{\text{g}}}\right)$$, evaluated at $${R}_{{\text{g}}}$$, $$g\in \{1,\dots ,G\}$$ (the lower bound of each quintile defined by systemic advantage measure $$S)$$, and equitability indices $${Q}_{{\text{t}}}$$ for our simulated ORCT. In Fig. 2a, concentration curves for a subset of interventions (chosen based on their potential optimality, described further below) illustrate that some interventions’ concentration curves lie predominantly below the 45° line, indicating that a higher proportion of health gains are expected to go to individuals with high relative advantage ($${\text{A}}$$ and $${\text{AB}}$$); some lie predominantly above the 45° line, indicating that a higher proportion of health gains are expected to go to individuals with low relative advantage ($${\text{D}}$$, $${\text{BC}}$$, and $${\text{BCD}}$$); and some reflect approximate neutrality of the distribution of gains with respect to relative advantage ($${\text{ABC}}$$ and $${\text{ABCD}}$$).

Figure 2b shows the equitability indices ${Q}_{{\text{t}}}$ for each intervention, aggregating the area between each intervention’s concentration curve and the 45° line, ordered from interventions with gains most concentrated among the relatively advantaged on the left to those with gains most concentrated among the relatively disadvantaged on the right (interventions $Min$ and $C$ are excluded as their estimated expected outcome gains were approximately zero). As expected, interventions including components A and B tend to most favor the relatively advantaged, while interventions including components C and D tend to most favor the relatively disadvantaged.

#### Step 3: Strategically Balancing Net Health Value and Equitability

We now return to the full set of interventions $t=\{1,\dots ,T\}$; importantly, we do not restrict decision-making at this point to the alternative interventions that fall on the original value efficiency frontier, because it is possible that an intervention that is dominated in terms of value efficiency may be favorable enough in terms of equity to be considered optimal when equitability is a key criterion. From the estimated group-specific expected outcomes ${\widehat{Y}}_{{\text{k}},{\text{t}},{\text{g}}}, k\in \left\{1,\dots K\right\}, g\in \{1,\dots ,G\}$, we obtain the health value gain concentration index ${H}_{{\text{t}}}$ for each intervention $t=\{1,\dots ,T\}$ by applying Eqs. (2) and (3).

**Fig. 3:**
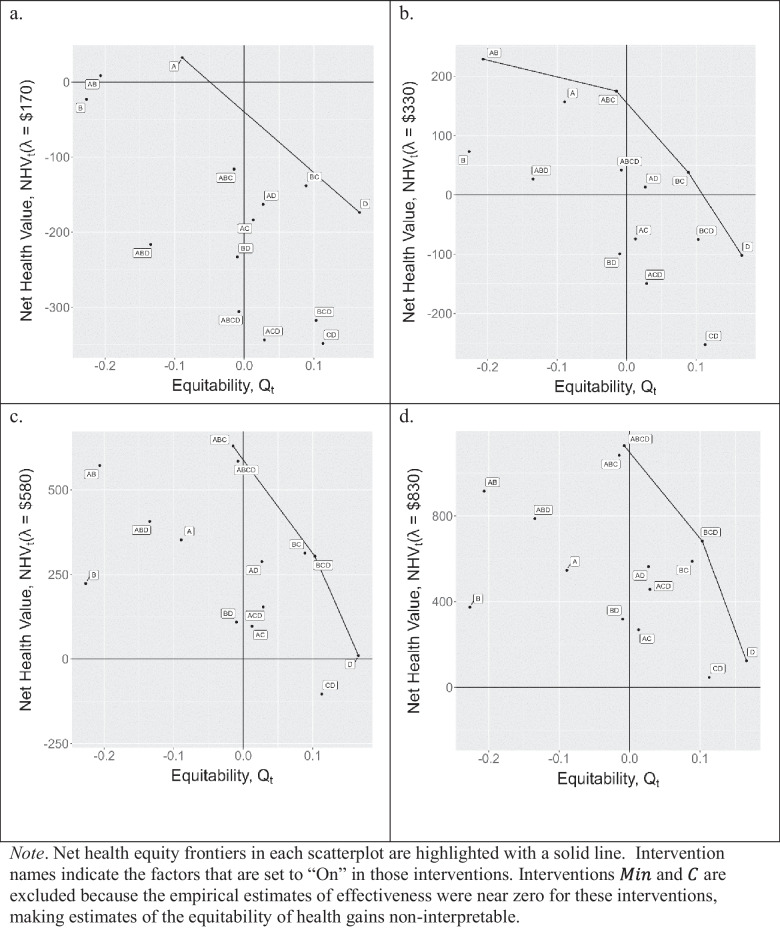
Equitability-net-health-value scatterplots based on the simulated factorial ORCT at each level of willingness-to-pay (λ)

Figure 3 includes scatterplots of estimated intervention equitability and predicted net health value for each representative level of willingness-to-pay (Fig. 3a–d). In each panel, the upper-right convex hull of points (towards increasing net health value and equitability), depicted by a curve connecting the alternative interventions that lie along the hull, identifies a net health equity frontier representing the subset of alternative interventions that achieve the highest predicted net health value (assuming a willingness-to-pay $\lambda )$ for a given level of intervention equitability. Interventions not on the upper-right convex hull are dominated in the sense that other interventions are expected both to achieve higher net health value overall and to avoid concentrating valued health outcome gains among individuals who are relatively advantaged.

At the lowest willingness-to-pay (*λ* = $170), the net health equity frontier (Fig. 3a) identifies just two contenders for the optimized intervention: intervention A and intervention D. Intervention A is associated with positive predicted net health value but negative equitability, whereas intervention D is associated with positive equitability but negative predicted net health value. The intuition here is that the decision-maker’s willingness-to-pay is related to the opportunity cost incurred when reallocating resources from other uses to pay for intervention D. In this case, the lowest willingness-to-pay implies the highest opportunity costs. While implementing intervention D would benefit individuals receiving the intervention, other individuals in the population who lose access to reallocated resources will suffer greater health loses than the gains experienced among those who use intervention D, thereby causing net population health to fall. There may be no easy answer for what is optimal in this case, since at this low willingness-to-pay there is a clear tradeoff between net health value and equitability. Notably, intervention A was a member of the original value efficiency frontier from the earlier decision-making that balanced effectiveness and efficiency only (and it was a frontier member that was relatively less costly, which is why it emerges as a contender at this low willingness-to-pay). This makes sense, since that decision-making reflected net health value but not equitability.

At the next level of willingness-to-pay (*λ* = $330), the net health equity frontier (3b) contains four contenders: AB, ABC, BC, and D. Again, we recognize members of the original value efficiency frontier (AB and ABC), and we find that these are similarly situated in the upper left quadrant of the scatterplot, indicating positive predicted net health value but negative equitability. Now the original frontier members that emerge as contenders are more costly (AB and ABC versus intervention A), reflecting the somewhat higher willingness-to-pay. Here too, Intervention D has a desirable positive equitability, but an undesirable negative predicted net health value. Intervention BC, meanwhile, is situated in the upper right quadrant, indicating positive predicted net health value and positive equitability. There is still a tradeoff associated with choosing intervention BC; intervention BC has less predicted net health value than interventions AB and ABC, but better equitability. If intervention BC is chosen as the optimized intervention, some net health value has been traded for better equitability in the strategic balancing.

At willingness-to-pay *λ* = $580 the net health equity frontier (Fig. 3c) contains three contenders: ABC, BCD, and D. The original frontier member, ABC, is once again situated in the upper left quadrant but just barely; intervention ABC has positive predicted net health value and an equitability that is less than zero but not by much. Intervention BCD is situated in the upper right quadrant, with positive predicted net health value and positive equitability. This makes BCD a particularly good option for the optimized intervention, though as usual the degree to which BCD is preferred over ABC depends on how much the investigator cares about equitability versus net health value. Interestingly, at this willingness-to-pay, intervention D is also situated in the upper right quadrant, but just barely, with very positive equitability and a predicted net health value that is just greater than zero. As before, choosing D would mean a strong preference for equitability over net health value—though, at this willingness-to-pay, intervention D has some small positive predicted net health value. The change in quadrants for intervention D serves as a reminder that net health value is a function of not only effectiveness but also efficiency, and efficiency depends on willingness-to-pay. At lower willingness-to-pay, the opportunity costs associated with choosing intervention D, or the other health outcomes foregone by reallocating resources from other programs to pay for intervention D, mean that the net health value is negative overall, despite the benefits for those who have relatively less advantage. When the willingness-to-pay increases, these opportunity costs are not perceived to be so steep, and the net health value for intervention D therefore increases.

Finally, at the highest willingness-to-pay (*λ* = $830) the net health equity frontier (Fig. 3d) contains three contenders: ABCD, BCD, and D. It is at this high willingness-to-pay that we see the intervention that maximizes overall effectiveness, ABCD, emerge as a contender. This alternative intervention, like the other original frontier members, has very positive predicted net health value but negative equitability (albeit slightly). Intervention BCD, meanwhile, is squarely situated in the upper right quadrant, with positive predicted net health value and positive equitability, and intervention D is again in the upper right quadrant, this time with predicted net health value that is more positive still (due to another increase in willingness-to-pay) and, as usual, very positive equitability. At this high willingness-to-pay, the choice of optimized intervention can be summarized as a decision among the following: ABCD, an intervention that is high in predicted net health value but has slightly negative equitability, meaning that the intervention tends to favor the already-advantaged; BCD, an intervention that trades some net health value for positive equitability, while remaining positive in predicted net health value; and D, an intervention that trades even more net health value (i.e., most of the predicted net health value offered by ABCD) for more positive equitability, or for benefits that tend to favor those who have relative less advantage.

At each willingness-to-pay, the ultimate selection of an optimized intervention from among the identified contenders depends on the investigator’s preferences (e.g., for equitability versus net health value). For a more technical demonstration of this, see the Technical Appendix.

## Discussion

MOST is a principled approach to optimization of interventions for prevention of health disorders, treatment of disease, and enhancement of well-being. Previous literature on MOST has defined optimization as a strategic balance of intervention effectiveness, affordability, scalability, and efficiency. In this article, we have extended the concept of optimization to include intervention equitability, defined in terms of the extent to which health benefits for an intervention are distributed evenly (versus concentrated among those who are already relatively more advantaged), as an additional element in this strategic balance. We used a hypothetical example to demonstrate how MOST can be used to illuminate the tradeoffs necessary to achieve an acceptable level of equitability when optimizing an intervention. We showed how different interventions may emerge as optimal (i) when equitability is included as a decision-making criterion versus when it is not; (ii) depending on how strongly decision-makers care about equitability versus other criteria; and (iii) under different levels of willingness-to-pay, because the tradeoffs between equitability and other criteria tend to be more severe when there is lower willingness-to-pay.

## Design of Research when Optimizing with Health Equity in Mind

Everything we have presented here has assumed the ORCT has been based on a sample sufficiently representative to enable valid conclusions to be drawn about equitability across the relevant subpopulations. In other words, when conducting an ORCT with the intention of optimizing for equitability, it is critical to attend not only to the internal validity of the experiment but to the external validity as well. If individuals with relatively less advantage are not adequately represented, however “advantage” may be defined, it is unlikely that the estimates of equitability will be accurate and, by extension, unlikely a sound decision can be made about the composition of the optimized intervention. Anwuri et al. (2013) outlined an approach for promoting recruitment and retention of members of minoritized groups. As they pointed out, it may be necessary to oversample some minoritized groups to have sufficient information for decision-making purposes. The present article also assumes that disadvantage is measured with adequate reliability and validity; if disadvantage is not measured accurately, misleading conclusions may be drawn. The measurement of systemic advantage versus disadvantage presents a set of theoretical and methodological challenges (Galobardes et al., 2006) that have been noted for some time and are beyond the scope of this paper to address.

In the present simulation, we assumed not only that our sample was sufficiently diverse and sampled uniformly across strata defined by relative advantage, but also that it was quite large (*N* = 800). We emphasize that the work to test robustness of these methods to variations in sample size has not yet been done. Our purpose in this simulation was to present an initial concept—and therefore, to ensure signal versus noise; we erred in the direction of a larger versus smaller sample size in order to provide a clear demonstration of tradeoffs. A critical next step will involve determining what sample sizes—and maybe what sample compositions—are necessary to consider intervention equitability as a criterion in intervention optimization. It is possible that any methodology that seeks to address differences in the effectiveness of complex interventions as a function of relative advantage will require samples that are adequately-sized (i.e., large) and representative enough to identify differences. This may, in turn, require larger investments in research.

## Equitability and Continual Optimization

Continual optimization is one of the fundamental principles of MOST (Collins, 2018). This principle states that MOST offers the opportunity to work iteratively toward making an intervention better and better. Unlike the classical treatment package approach, MOST enables the investigator to see clearly what the next steps are to improve an intervention, because the ORCT shows which components are working well and should be retained, and which are working poorly (or even are counterproductive) and should be revised or replaced. Thus, successive ORCTs can be undertaken to assess the performance of new components intended to make an intervention more equitable. Then, repeated optimization based on each successive ORCT can work toward a more and more equitable intervention, while at the same time balancing effectiveness, affordability, scalability, and efficiency. This is potentially one way to make material progress toward not only health equity, but greater public health impact.

## Limitations and Future Directions

We began this article by stating that it represents an initial foray into incorporating health equity into intervention optimization and that much remains to be done. In presenting our ideas, we made three simplifying assumptions, each of which suggests an intriguing area for future research.

First, our hypothetical example involved a single outcome variable, whereas many interventions target more than one outcome variable. DAIVE has been applied to situations involving multiple outcomes, based on Bayesian decision analysis and using a value function to combine several outcomes (Strayhorn et al., 2023a, b). A variety of value functions have been proposed that can be used to reflect different decision-maker preferences, for example, preferences about the relative importance of the outcomes. Because the simulation we present involved a single outcome variable, we have not described how these more complex value functions will be used in applications of DAIVE that incorporate equitability as a criterion, but the extension is relatively straightforward.

Second, we presented a hypothetical fixed intervention, in which all participants are offered the same treatment. Much attention in the intervention optimization field has been paid to adaptive interventions (e.g., Almirall et al., 2018a, b; Nahum-Shani et al., 2018a, b). Adaptive interventions are varied in a principled fashion in response to characteristics of the individual or environment, representing a more individualized and, potentially, more effective and efficient approach to prevention and treatment. DAIVE has not yet been extended to decision-making based on the results of ORTCs such as the sequential multiple-assignment randomized trial (SMART; Almirall et al., 2018a, b) and the micro-randomized trial (MRT; Qian et al., 2022) that are used for optimization of adaptive interventions. We also do not address the possibility that decision-makers would consider delivering different interventions, composed of different components, to different subgroups of a population, defined in terms of relative advantage. Using results like those we present here to inform such decision-making is an interesting idea but one that presents complex ethical dilemmas that would require further careful attention.

Third, given the important role of multi-level interventions across public health and education, it is a serious limitation that the ideas we presented are so far limited to single-level interventions. Consider the hypothetical intervention presented in this article. One or more of the components could be delivered in a group setting, so that participants are nested within groups, and random assignment at the group level would be necessary. Or suppose the participants in the hypothetical intervention are nested within medical clinics, and that in addition to the components aimed at participants, the intervention includes a component to reduce HIV-related stigma, aimed at the clinic level. This might require a random assignment at both the clinic and individual level. There has been some work on multilevel ORTCs (e.g., Dziak et al., 2012; Nahum-Shani et al., 2018a, b), but this work has pertained primarily to situations in which although there is a multi-level structure, the intervention is single-level and inference is made solely at a single level. More work is needed on multilevel ORCTs for situations in which the intervention to be optimized is multi-level. DAIVE has not yet been extended to the results of multilevel ORTCs; this is a critical next step.

## Conclusions

In this article, we have suggested that MOST provides a principled framework for designing equitable interventions, in other words, interventions that reduce health disparities and promote health equity. In our view, if the ultimate goal for an intervention is high public health impact for all, effectiveness and equitability must be balanced strategically against each other and against key resource considerations, including affordability, scalability, and/or efficiency. Achieving this complex strategic balance can require difficult tradeoffs. For example, as we have demonstrated, sometimes to achieve equitability it may be necessary to choose either sacrificing some overall effectiveness or increasing willingness to pay. MOST can be used to help illuminate the tradeoffs that arise when seeking to develop equitable interventions, so that these tradeoffs can be faced unsparingly, considered thoughtfully, and made with transparency.

Much remains to be done to expand the utility of MOST in optimizing interventions with equitability in mind. Nevertheless, we are optimistic that MOST can play a role in increasing intervention equitability, and, by extension, reducing health disparities and enhancing health and well-being for all.

## Data Availability

Simulated data can be made available upon request via the corresponding author.
